# Complete genome sequence of *Roseinatronobacter* sp. strain S2, a chemolithoheterotroph isolated from a pH 12 serpentinizing system

**DOI:** 10.1128/MRA.00288-23

**Published:** 2023-08-16

**Authors:** Leah R. Trutschel, Annette R. Rowe, Joshua D. Sackett

**Affiliations:** 1 Department of Biological Sciences, University of Cincinnati, Cincinnati, Ohio, USA; University of Delaware College of Engineering, Newark, Delaware, USA

**Keywords:** serpentinization, alkaliphile, sulfur oxidation, chemolithoheterotroph, Ney Springs

## Abstract

Here, we report the complete genome sequence for *Roseinatronobacter* sp. S2, a sulfur-oxidizing heterotroph isolated from a serpentinizing system in Northern California. The S2 genome is 4.4 Mb and contains 4,570 protein-encoding genes. This organism contains the genes necessary for sulfur species oxidation and complete ethylmalonyl and pentose phosphate pathways.

## ANNOUNCEMENT

Serpentinizing systems are hyperalkaline (pH >10) environments characterized by the presence of abiogenic hydrogen and methane (1 - 5). Ney Springs is a pH 12–12.5 marine-like spring characterized by high amounts of sulfide, ammonia, and methane (6). Many abundant microorganisms in this spring are putative sulfur oxidizers belonging to the *Paracoccaceae* (formerly *Rhodobacteraceae)* (6). *Roseinatronobacter* sp. S2 is one of the first sulfur-oxidizing isolates obtained from a serpentinizing system (6); its completed genome provides insight into the adaptations and metabolism of alkaliphilic chemolithoheterotrophs.

S2 was isolated from Ney Springs in Mt. Shasta, California, on minimal media agar plates containing 20 mM polysulfide and 10 mM acetate (6). S2 was cultivated aerobically in liquid minimal media containing 20 mM thiosulfate and 10 mM acetate. Detailed media instructions are found here: dx.doi.org/10.17504/protocols.io.bqjgmujw. S2 was incubated at room temperature for 5 days to achieve approximate maximum turbidity as determined by a previous growth curve (6). DNA was extracted using a Qiagen DNeasy PowerSoil Kit and quantified using a Qubit fluorometer (ThermoFisher Scientific, USA). All sequencing was done from a single DNA prep. Nanopore libraries were prepped with the Native Barcoding 24 V14 Kit (Oxford Nanopore Technologies, Oxford, UK) and sequenced using a R10.4.1 flow cell (FLO-MIN114) under high-accuracy mode (280 bp/s) with a MinION-sequencing device. Basecalls were made with Guppy v.6.4.6, and reads with quality scores <7 were removed (7). Illumina library prep and sequencing were conducted at SeqCenter LLC (Pittsburgh, PA, USA). Briefly, libraries were prepared with the Illumina DNA Prep Kit, barcoded with 10 bp unique dual indices, and sequenced on an Illumina NovaSeq (2 × 150 sequencing). Demultiplexing, quality control, and adapter trimming were performed with bcl-convert (v.4.0.3). Nanopore sequences >2,000 bp were quality filtered with Filtlong (v.0.2.1) (8), and the resulting worst 10% of read bases were removed. Filtered long reads were assembled with Flye (v.2.9.1) (9). Illumina reads were quality filtered with FastQC (v.0.12.1) (10) with all reads passing with a quality score >Q30. Short reads were aligned to the assembly with Burrows-Wheeler Aligner (v.0.7.17) (11), and the assembly was polished with Pilon (v.1.24, -fix all) (12). Four rounds of polishing were performed. Quality assessment and genome statistics were determined using QUAST (v.4.4) (13) and CheckM (v.1.0.18) (14) within the KBase wrapper (15). Taxonomic classification was done with GTDB-tK (V. 2.2.5) (16). The genome was annotated with the NCBI Prokaryotic Genome Annotation Pipeline v6.5 (17). Default settings were used for all programs unless noted.

The final assembly size for the genome was 4,445,845 bp, and it has a GC content of 59.5%. The assembly was composed of six circular contigs, potentially chromids or plasmids, as often seen in members of the *Paracoccaceae* (18). GTDB-Tk classified the assembly as *Roseinatronobacter* with an average nucleotide identity of 84.66% with the nearest neighbor *Roseinatronobacter* sp. 017510335 (alignment fraction = 0.747). S2 is predicted to contain the ethylmalonyl pathway, which has been used as a way to utilize acetate in bacteria lacking isocitrate lyase (19). S2 contains SoxBCDYZ, Sqr, and FccB and is capable of thiosulfate oxidation to sulfate *in vitro* (6).

**TABLE 1:** Summary of genome statistics

Parameter	Finding
Illumina reads accession no.	SRR23870722
MinION reads accession no.	SRR23870721
Genome assembly accession no.	CP121113, CP121114, CP121115, CP121116, CP121117, CP121118
Assembly N_50_ (bp)^*a*^	3,479,920
Nanopore N_50_ (bp)^*b*^	11,120
Assembly size (bp)	4,445,845
Nanopore read length (bp)^*b*^	686,780,239
Illumina read count (seqs)	2,475,882
G + C content (%)	59.5
Estimated genome completeness (%)^*c*^	98.47
Estimated genome contamination (%)^*c*^	1.21
Estimated Nanopore coverage of largest contig (Genome) (×)^*b*^	155
Estimated Illumina coverage (×)	152
No. of contigs	6
Contig_1 length (chromosome) (bp)	3,471,855
Contig_3 length (bp)	611,552
Contig_5 length (bp)	166,770
Contig_4 length (bp)	136,430
Contig_6 length (bp)	33,793
Contig_2 length (bp)	15,307
No. of protein-coding genes	4,570
No. of tRNAs	47
No. of rRNA operons	3

^*a*^ Determined with Quast (13).

^*b*^ Determined with Flye (9).

^*c*^ Determined with CheckM (14).

## Data Availability

The raw sequencing data were submitted to the NCBI Sequence Read Archive, and the genome assembly was submitted to GenBank under the accession numbers listed in Table 1.
